# circACTN4 promotes breast cancer cell cycle progression and oncogenesis via c-MYC induced histone H4 acetylation

**DOI:** 10.32604/or.2025.061721

**Published:** 2025-06-26

**Authors:** KEFAN LIU, XIAOSONG WANG, XIN YANG, BOWEN SHI, LEI XING, JUNXIA CHEN

**Affiliations:** 1 Department of Cell Biology and Genetics, Chongqing Medical University, Chongqing, 400016, China; 2 Department of Breast and Thyroid Surgery, The First Affiliated Hospital of Chongqing Medical University, Chongqing, 400016, China

**Keywords:** circACTN4, Breast cancer, Histone H4, Acetylation, Myc

## Abstract

**Background:**

Accumulating studies have shown the important role of circular RNAs (circRNAs) in the oncogenesis and metastasis of various cancers. We previously reported that circACTN4 could bind with FUBP1 to promote tumorigenesis and the development of breast cancer (BC) by increasing the expression of MYC. However, its exact molecular mechanism and biological function have not been fully elucidated.

**Methods:**

Here, Circular RNA microarray analysis was conducted in 3 pairs of BC and paracancerous tissues. The expression of circACTN4 in BC cells and tissues was detected via reverse transcription‒quantitative PCR (RT‒qPCR). Cell Counting Kit-8 (CCK-8), 5-ethynyl-2-deoxyuridine (EdU), transwell migration, and invasion assays were performed to further detect the biological functions of circACTN4 in BC cells. Xenograft models were used to investigate the *in vivo* role of circACTN4. Fluorescence *in situ* hybridization, Chromatin immunoprecipitation (ChIP)‒qPCR, coimmunoprecipitation, fluorometric, western blot, and rescue experiments were performed to explore the mechanism of circACTN4.

**Results:**

Our results revealed that circACTN4 was highly expressed in BC cells and tissues. The upregulated expression of circACTN4 was significantly related to the T stage and TNM stage and poor prognosis of patients with BC. circACTN4 was located primarily in the nucleus of BC cells. Upregulation of circACTN4 significantly increased the proliferation, invasion, and growth of BC cells, whereas the downregulation of circACTN4 exerted the opposite effects and induced G1/S cell cycle arrest. Mechanistically, we showed that circACTN4 could upregulate the expression of MYC and that MYC might interact with TIP60 histone acetyltransferase to increase the recruitment of TIP60 to MYC target genes and histone H4 acetylation (AcH4), thus promoting the progression of the breast cancer cell cycle and tumorigenesis.

**Conclusion:**

Taken together, our findings reveal for the first time a new mechanism by which circACTN4 could promote oncogenesis and the development of BC by increasing the AcH4 of MYC target genes via TIP60. Therefore, circACTN4 could be a novel target for BC diagnosis and remedy.

## Introduction

Breast cancer stands as the preeminent female-exclusive malignant neoplasm on a global scale, ranking first in incidence among women, and plays a dominant role in accounting for cancer-related deaths in women on a global scale. Although important progress has been made in diagnosis and therapy in decades, the incidence rate of BC has increased significantly. Breast cancer accounts for about 30% of cancer cases and 15% of female cancer deaths [1,2]. Thus, there is an imperative need to identify novel promising molecular targets for the diagnosis and therapeutic intervention of cancer.

Circular RNAs (circRNAs) are a kind of closed circular single-stranded RNA without a 5′ end cap and 3′ poly A tail formed by back splicing of linear precursor RNAs. Owing to their circular characteristics, circRNAs are more refractory to exonuclease degradation and thus demonstrate a substantially higher level of stability than canonical linear RNA [3]. A growing body of well-documented research findings has substantiated that some circRNAs are dysregulated in various tumors. CircRNAs are implicated in the occurrence, development, and immune escape of tumors, suggesting their potential functional importance.

CircRNAs can exert their roles in absorbing miRNAs, interacting with RNA binding proteins (RBPs), and translating proteins [4–6]. Certain circRNAs have been shown to be crucial in breast cancer development. As an illustration, Circ_0000520 promotes TNBC progression via the miR-1296/ZFX axis [7]. In addition, EIF6-224 aa, encoded by circ-EIF6, stabilizes MYH9 and activates the Wnt/beta-catenin pathway to promote TNBC progression [8]. Furthermore, circ_0001667 increases BC cell proliferation and angiogenesis by regulating the miR-6838-5p/CXCL10 axis [9]. Our previous study demonstrated that circACTN4 might exhibit competitive binding and affinity towards FUBP1 to impede the interaction of FUBP1 and FIR, consequently, it facilitates the transcriptional process of MYC and the progression of breast neoplasia [10]. However, the detailed molecular mechanism underlying the interaction between MYC and circACTN4 in BC remains to be further explored.

The nucleosome around the core histones H2A, H2B, H3, and H4 is the first level of DNA packaging in eukaryotes. In recent years, histone acetylation and deacetylation have been shown to exert substantial influences on the transcriptional regulating of all eukaryotes. Acetylation levels are modulated by various histone acetyltransferases (HATs) and deacetylases (HDACs), which are recruited into promoters by activators and repressors to mediate changes in chromatin structure and regulate gene expression. TIP60 histone acetyltransferase is the key enzyme that catalyzes H4 acetylation, which is implicated in chromatin packaging, transcription activation, and DNA repair [11–13]. It has been demonstrated that the TIP60 HAT complex can be recruited to MYC target genes by MYC. MYC induces the acetylation of histone H4 at some target loci via TIP60 in response to mitogenic signals [14–16]. However, the mechanism by which circRNAs regulate H4 acetylation and transcription of MYC target genes has not yet been reported.

In the current research, we further elucidated the cellular expression status and molecular bioactivities of circACTN4 in BC. Importantly, we also demonstrated that circACTN4 upregulates the gene expression level of MYC and promotes AcH4 of the CCNE1 (cyclinE1) and CDK4 (cyclin-dependent kinase) genes, which led to increases in the gene-expression magnitude of CCNE1 and CDK4, cell cycle progression, and tumorigenesis in the breast. The study reveals a hitherto unreported mechanistic pathway governing the advancement of breast cancer and suggests that circACTN4 has the potential to become a new biomarker for the diagnosis and therapy of breast cancer.

## Materials and Methods

### Clinical tissue specimens

The 50 clinical tissue specimens used in this study, including BC and pertumoral tissues, were obtained from breast cancer patients who received mastectomies at the First Affiliated Hospital of Chongqing Medical University (Chongqing, China). The postoperative specimens were promptly cryopreserved in liquid nitrogen and maintained in this cryogenic state until they were removed for subsequent experiments. Before the surgical procedure, each patient executed an informed consent form, and the Ethics Committee of the First Affiliated Hospital of Chongqing Medical University approved the collection of all the specimens in the study (No. 2022-K228).

### Cell culture

The normal breast epithelial cell lines MCF-10A, MDA-MB-231, and MDA-MB-453 BC were all preserved in our laboratory. Prior to utilization, all cells underwent a comprehensive examination to detect the presence of mycoplasma. The cells were cultured in DMEM (Saimike Bio, SMK200.01, Hangzhou, China) containing 10% fetal bovine serum (Tianhang Bio, 13011-8611, Hangzhou, China). MCF-10A cells were cultured in MEBM BulletKit medium (Thermo Fisher Scientific, M171500, Walthem, Massachusetts, USA). 1% penicillin/streptomycin was added to all media. All the cell cultures were maintained under a controlled environment of 37°C and 5% CO_2_.

### Microarray analysis

Total RNA from 3 pairs of BC and matched normal tissues were isolated via TRIzol reagent (Takara, 9180Q, Dalian, China), quantified, and digested via RNase R to remove linear RNAs for enrichment of circRNAs. Sample preparation and microarray hybridization were conducted in compliance with the standard SOP (standard operating procedure) of Arraystar provided by KangChen Biotech (Shanghai, China). The Arraystar Human circRNA Array V2 was utilized for microarray analysis of circRNAs.

### Fluorescence in situ hybridization (FISH) and in situ hybridization (ISH)

We designed a specific Cy3-labeled probe for circACTN4 (5’Cy3-GCCGTGAAGGTCCTCTGCATC-3’Cy3), which was synthesized by Geneseed, Guangzhou, China. The cell nuclei were counterstained with 4,6-diamidino-2-phenylindole (DAPI) (RiboBio, C10910, Guangzhou, China). Images were obtained by means of an Olympus BX51 fluorescence microscope (Tokyo, Japan). TMA (Tissue microarray) was purchased containing 100 paraffin-embedded tissue specimens from Outdo Biotech (Shanghai, China). ISH was utilized to determine the expression level of circACTN4 within BC tissues. The samples were hybridized by a specific probe labeled with digoxin (Geneseed, Guangzhou, China). The tissues were subjected to an incubation process with AP-conjugated anti-digoxin antibody (Roche, 11093274910, Basel, Switzerland) at 4°C for an overnight period, then stained by NBT/BCIP (Roche, B1911, Basel, Switzerland). Levels of circACTN4 were determined and analyzed. The score of ISH staining is calculated. ISH score ≤ 8 indicated low expression, while >8 was considered high expression. The positive staining intensity (negative = 0; weak = 1; moderate = 2; strong = 3) was multiplied with the proportion of cells exhibiting positive staining (0, <10% = 0; 10%–25% = 1; 26%–50% = 2; 51%–75% = 3; >75% = 4).

### Plasmid construction, RNAi, and transfection

The full length of circACTN4 was cloned into the pLC5-ciR vector to design a circACTN4 overexpression vector. The control was a mock vector without the circACTN4 sequence. To knock down circACTN4, we synthesized siRNAs targeting circACTN4 (si-circ#1, si-circ#2) and NC siRNAs (Geenseed, Guangzhou, China). The human circACTN4 gene was successfully ligated into the lentiviral vector CV146. si-circ#1 was inserted into the lentiviral vector GV344 by Genechem (Shanghai, China). HEK293T cells were infected with each group of lentiviral vectors, and the viral supernatant was removed 72 h later for the animal assay. We inserted the human MYC gene into the vector GV658 to construct an overexpression vector via GeneChem (Shanghai, China), and siRNAs targeting MYC were produced by RiboBio (Guangzhou, China). Stably transfected cells were selected with purocycin (Labgic, BL528K, Beijing, China). The transfection experiments were implemented by Lipofectamine 2000 (Invitrogen, 11668030, Carlsbad, CA, USA). The nucleotide sequences of the siRNAs are comprehensively documented in Table 1 for reference.

**Table 1:** Sequences of siRNAs

Definition	sequences
si-circ#1	ATGCAGAGGACCTTCACGG
si-circ#2	CAGAGGACCTTCACGGCAT
si-NC	TTCTCCGAACGTGTCACGT
si-MYC#1	GAGGAGACATGGTGAACCA

### CCK-8 and EdU experiments

A density of 2.0 × 10^3^ BC cells per well was precisely seeded in triplicate into 96-well plates (Labgic). The cells were incubated with CCK-8 (Hanbio, HB-CCK-8-500, Shanghai, China) at the indicated times for 1 h. The absorbance value of a wavelength of 450 nm was measured and documented at the specified time points. We inoculated BC cells into 24-well plates (Labgic) for 5-ethynyl-2′-deoxyuridine (EdU) assays. The cells were stained with EdU reagent (RiboBio, C10310-1, Guangzhou, China) and 4’,6-diamidino-2-phenylindole (DAPI) (RiboBio, C10310-1, Guangzhou, China) to observe the cell propagation images under a fluorescence (Thermo Fisher Scientific, IQLAADGAAGFAQPMBJG, USA).

### Transwell migration and invasion assays

For the purpose of conducting the migration assay, a volume of 500 μL of complete medium was precisely dispensed into the lower chamber of the Transwell system (Labgic, Beijing, China), and then 2 × 10^4^ BC cells were added to the upper chamber and incubated in an incubator with 5% CO_2_ (Thermo Fisher Scientific, 4131, USA) for 24 h. The cells were fixed with 75% ethanol and stained with a crystal violet solution (BeyotimeBio, C0121, Shanghai, China). In the context of the invasion assay, a 50-μL aliquot of Matrigel (Corning, 356234, Corning, NY, USA) diluted with serum-deprived medium was added to the upper chamber of the Transwell system, which was incubated at 37°C for a duration of 1 h. The remaining steps were similar to those of the migration experiment.

### qRT‒PCR assay

We extracted total RNA from cells and tissues with Trizol (Takara, 9180Q, Dalian, China) in accordance with the standard protocol. The PARIS™ Kit (Life Technologies, AM1921, Austin, TX, USA) was utilized to isolate RNAs from the nucleus and cytoplasm of cells. The reverse transcription experiment was executed via a PrimeScript RT kit (Sparkjade, AJ0210, Jinan, China), and qRT‒PCR was carried out by TB green premix Ex-Taq (Takara, Dalian, China). The relative expression level was computed via the 2^−ΔΔCT^ method, a widely recognized qPCR quantification approach. Primer sequences are detailed in Table 2.

**Table 2:** Sequences of primers

Gene	Primer sequences
circACTN4	F:5’-AAGGACGACCCTGTCACCAA-3’
	R:5’-CCGTGAAGGTCCTCTGCAT-3’
GAPDH	F: 5’-GAAGGTGAAGGTCGGAGTC-3’
	R: 5’-GAAGATGGTGATGGGATTTC-3’
MYC	F: 5’-GGCTCCTGGCAAAAGGTCA-3’
	R: 5’-CTGCGTAGTTGTGCTGATGT-3’
CDK4	F: 5’-TCTGGACACTGAGAGGGCAATC-3’
	R: 5’-GAAATGGGAAGGAGAAGGAGAAGC-3’
CCNE1	F: 5’-GTCCTGGATGTTGACTGCCTTG-3’
	R: 5’-GTTCTCTATGTCGCACCACTGATAC-3’

### Cell cycle analysis

With the aim of investigating the cell cycle, a total of 1 × 10^6^ viable cells were collected and subsequently subjected to a meticulous analysis of the cell cycle, the cells were subjected to fixation in 70% ethanol at a temperature of 4°C for an overnight incubation period, stained with PI (propidium iodide, P4170, Darmstadt, Germany), and then detected via flow cytometry (Becton Dickinson FACS Calibur, 342973, Franklin Lakes, NJ, USA).

### Western blotting

For the extraction of proteins, cells were subjected to lysis in ice for 25 min using RIPA buffer (Solarbio, R0010, Beijing, China). Subsequently, ultrasonic treatment was performed to disrupt the cell membranes and facilitate the release of proteins. Following centrifugation, the supernatant containing proteins was carefully collected. Thereafter, the bicinchoninic acid (BCA) working solution (Labgic, BL2440B, Beijing, China) was prepared. A volume of 200 μL of the BCA working solution was pipetted into each well of a 96-well microplate. Then, 19 μL of phosphate-buffered saline (PBS) (Labgic, BL2440B, Beijing, China) and 1 μL of the extracted protein sample were added to the wells and thoroughly mixed. Each sample was assayed in triplicate. The microplate was incubated at 37°C for 30 min and subsequently cooled to room temperature. The optical density from the sample wells was determined at a wavelength of 562 nm with the utilization of a microplate reader (Thermo Fisher Scientific, 1410101, USA). A standard curve was plotted based on the absorbance values of the protein standards with known concentrations. The protein concentration in each sample was determined via interpolating from the reference curve according to the measured absorbance values of the samples. The amount of extracted protein was determined via 10% SDS‒PAGE, and the separated protein was transferred to a PVDF (PolyVinylidene Fluoride) membrane (Bio-Rad, 1620264, Hercules, California, USA). After being blocked with 5% skim milk powder (Labgic, BS102, Beijing, China), the membranes were incubated in the presence of primary antibodies directed against MYC (1:2000, Proteintech, 10828-1-AP, Rosemont, IL, USA), CDK4 (1:1000, Proteintech, 11026-A-AP, Rosemont, IL, USA) and CCNE1 (1:1000, Proteintech, 11554-1-AP, USA) at 4°C overnight and then incubated with secondary antibodies (Proteintech, RGAR001, USA) at room temperature for 1.5 h. The bands were visualized via an enhanced chemiluminescence detection system (Thermo Fisher Scientific, A44241CFR, USA).

### Animal experiment

The research was approved by the Institutional Animal Care and Use Committee of Chongqing Medical University (IACUC-CQMU-2024-0200). The breeding and euthanasia methods of animals meet the requirements of scientific research ethics. The research design is scientifically based, and the project is approved to be implemented according to the predetermined plan.

Twelve female BALB/c nude mice at 4-week-old (the mice are reared in IVC breeding cages. The bedding materials and drinking water are sterilized for use. The drinking water bottles are replaced and feed is added twice a week) (SJA, Changsha, China) were selected, and 10^7^ cells infected with various lentiviruses were subcutaneously inoculated. The size of the tumors was monitored every seven days, and the volume was determined by applying the following formula: length × width^2^/2. After a 28-day experimental period, the nude mice were euthanized, and the tumors were obtained for immunohistochemical and pathological analyses.

### Immunohistochemical staining

Samples were first fixed using 4% paraformaldehyde. Subsequently, these fixed samples underwent the embedding process in paraffin. Finally, the paraffin-embedded samples were sectioned into slices for further analysis. The tissue sections were deparaffinized with xylene and immersed in graded alcohol, then washed three times. They were added with 0.3% H_2_O_2_, then treated with 0.5% TritonX-100 (BeyotimeBio, P0096, Shanghai, China) for 20 min, and subsequently treated with 10% sheep serum for 30 min. The samples were incubated at 4°C overnight with antibodies against MYC (1:50, Proteintech, 10828-1-AP), CCNE1 (1:50, Proteintech, 11554-1-AP), and CDK4 (1:50, Proteintech, 11026-1-AP) and then incubated at 37°C with secondary antibodies (BeyotimeBio, A0277, Shanghai, China) for 1 h. The sections were dyed with DAB (BeyotimeBio, P0202, Shanghai, China) and hematoxylin (BeyotimeBio, C0107, Shanghai, China), and images were taken under a microscope (Leica, DM750P, Witzlar, Germany). Protein expression levels were quantified and analyzed by a modified scoring method. The immunostaining intensity (0, negative; 1, weak; 2, moderate; 3, strong) was multiplied by the proportion of positively-stained cells (0, <10% = 0; 1, 10%–25%; 2, 26%–50%; 3, 51%–75%; 4, >75%) to calculate the score for IHC staining.

### Histone H4 acetylation detection

For the purpose of histone isolation, MDA-MB-231 cells were lysed, and histones were subsequently obtained through the utilization of an EpiQuik™ Total Histone Extraction Kit (Epigentek, OP-0006, Farmingdale, NY, USA). Total histone H4 acetylation was measured following the standardized protocol supplied by the manufacturer using an EpiQuik Total Histone H4 Acetylation Detection Fast Kit by Fluorometric (Epigentek, P-4033, Farmingdale, NY, USA). Briefly, AcH4 was bound to the assay wells coated with an anti-acetyl histone H4 antibody (CST, 13534, Beverly, MA, USA), after which a labeled detection antibody was added to the wells after washing, followed by the addition of a fluorescent development reagent. The fluorescence was measured with a fluorescence microplate reader (Thermo Fisher Scientific, VLBLATGD2, USA) at 530EX/590EM nm. The proportion of acetylated histone H4 exhibits a proportional relationship with the fluorescence intensity. The absolute quantity of acetylated histone H4 was ascertained through comparison with a standard control.

### Chromatin immunoprecipitation (ChIP) assay

Briefly, 1 × 108 MDA-MB-231 cells were fixed by formaldehyde, lysed, and then sonicated to produce DNA fragments of 200-1000 bp. The samples were subjected to an incubation process with anti-MYC (1:100, CST, 18583, Beverly, MA, USA), anti-acetyl-histone H4 (1:50, CST, 13534, Beverly, MA, USA) or IgG (CST, 2729, Beverly, MA, USA) antibodies at 4°C for 14 h. ChIP-grade protein A/G magnetic beads (Thermo Fisher Scientific, 26156, USA) and antibody‒chromatin complex were incubated at 4°C for 2 h. DNA was eluted, purified, and then subjected to qRT‒PCR. The primer sequences for ChIP-qPCR have been documented in Table 2 for reference.

### Coimmunoprecipitation (co-IP)

Antibodies against MYC (1:100, CST, 18583, Beverly, MA, USA) and TIP60 (1:100, CST, 12058, Beverly, MA, USA) or IgG (CST, 2729, Beverly, MA, USA) were covalently linked by magnetic beads at 4°C for 4 h. MDA-MB-231 cells were lysed in IP lysis buffer via the PierceTM Classic Magnetic IP/Co-IP Kit (Thermo Fisher Scientific, 26149, USA). The magnetic beads coated with the antibody and cell lysates were mixed at 4°C overnight. The proteins specifically bound to the antibody-bead complexes were eluted with sample buffer and then conducted western blotting.

### Statistical analysis

Statistical analysis was implemented via GraphPad Prism 8.0 software (GraphPad Software, San Diego, CA, USA) and SPSS 21.0 software (IBM, SPSS, Chicago, IL, USA). All the experimental data are presented as the means ± standard deviations (SDs). Two-tailed Student’s *t*-test, one-way analysis of variance (ANOVA), and the $\chi^{2}$ test were used to compare the differences between two or more groups. All the quantitative data are the summary of 3 independent experiments or 3 independent measurements. The Kaplan–Meier plots and the log-rank test were used for survival analysis. Differences were considered statistically significant at *p* < 0.05.

## Results

### circACTN4 is highly expressed in BC and associated with clinical pathological features

**Figure 1:**
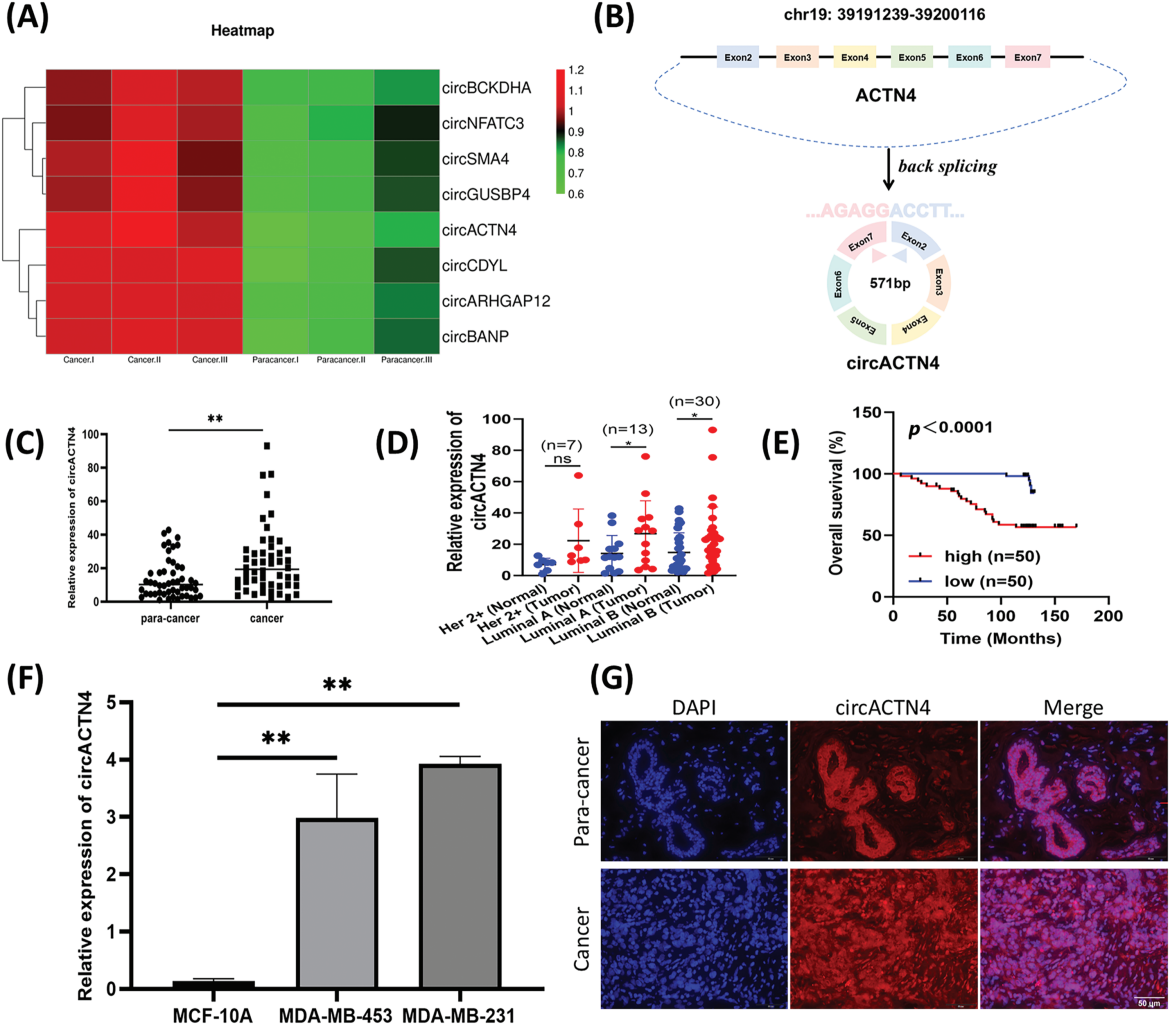
circACTN4 is upregulated in BC. (A) Microarray analysis shows representative differential expression of circRNAs between BC tissue and paired noncancer tissue. (B) Schematic diagram of circACTN4 formed by the cyclization of exon 2 to exon 7 of the ACTN4 gene. (C, D) The qRT-PCR was applied to quantify circACTN4 expression in 50 matched BC-paracancerous tissue pairs. (E) The patients with high expression of circACTB4 had a shorter overall survival than BC patients with low expression of circACTN4 by Kaplan-Meier survival analysis. (F) The qRT-PCR was employed for circACTN4 expression analysis in BC cells. (G) FISH detection revealed the subcellular distribution of circACTN4 and expression in BC tissue and paired noncancerous tissue (magnification, ×400, scale bar, 50 μm). The data are presented as the mean ± SD, **p < 0.01.

Microarray analysis revealed expression profiles of circRNAs differential expression between BC and paired noncancer tissues. Abnormal circACTN4 expression was observed in BC tissues (Fig. 1A). Based on the information provided by circBase, circACTN4 is located from exon 2 to exon 7 of the ACTN4 gene in human chromosome 19 and generates a 571-nucleotide covalently closed RNA molecule by back splicing (Fig. 1B). To explore the clinical value of circACTN4, the expression level of circACTN4 was detected in 50 sets of paired BC and adjacent noncancerous tissues via qRT‒PCR. The experimental findings demonstrated a statistically significant up-regulation of circACTN4 expression in BC tissues (Fig. 1C and D). Thereafter, we employed human tissue microarrays (TMAs) encompassing 100 breast cancer (BC) tissues to conduct an *in-situ* hybridization (ISH) assay for the detection of circACTN4 expression. Kaplan-Meier survival analysis unveiled that patient with high-level circACTN4 expression exhibited a notably shorter overall survival time compared to those with low-level circACTN4 expression (Fig. 1E). Furthermore, the qRT‒PCR results outcomes indicated that the expression level of circACTN4 was substantially elevated in BC cells than in the non-cancerous mammary epithelial cell line MCF-10A (Fig. 1F). The FISH assay revealed that circACTN4 showed a substantially enhanced transcriptional activity in BC tissues compared with paired noncancer tissues and that circACTN4 predominantly localizes within the nuclei of BC cells (Fig. 1G). Together, these results indicate that circACTN4 is highly expressed significantly in BC and could be a diagnostic and treatment marker for BC patients. Subsequently, we conducted an assessment of the associations between the expression level of circular RNA ACTN4 (circACTN4) and clinicopathological features. The result demonstrated that the level of circACTN4 expression exhibited a direct correlation with T stage (n = 50) and TNM stage (n = 50) (Table 3).

**Table 3:** Relationship between circACTN4 expression and clinical pathological characteristics in 50 BC patients

Characteristic	All cases	circACTN4		Chi-square	*p* value	
		low	high			
All cases		50	25	25		
Age	<53	26	14	12	0.321	0.571
	≥53	24	11	13		
Grade	I–II	38	20	18	0.439	0.508
	III	12	5	7		
T stage	T1	28	18	10	5.195	0.023^*^
	T2-3	22	7	15		
N stage	N0	31	14	17	0.764	0.382
	N1-3	19	11	8		
TNM stage	I	30	22	8	16.333	<0.001^***^
	II/III	20	3	17		

Note: *p < 0.05, ***p < 0.001.

### circACTN4 increases the proliferation and growth of BC cells in vitro and in vivo

**Figure 2:**
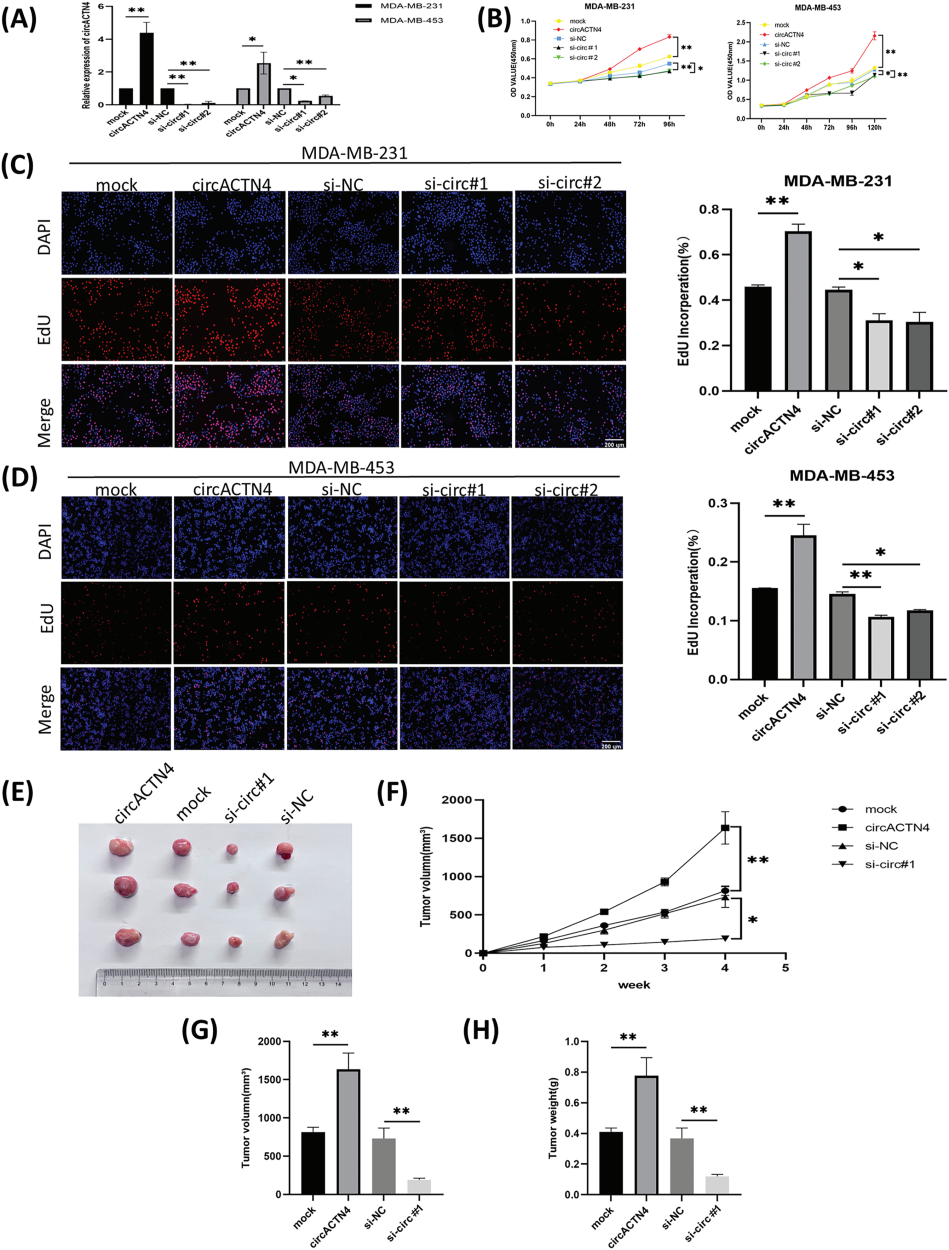
circACTN4 enhances the proliferation and growth of BC cells *in vitro* and *in vivo*. (A) The efficiency of the circACTN4 overexpression vector or siRNAs in BC cells was assessed via qRT‒PCR. (B) The viability of BC cells transfected with the circACTN4 overexpression plasmid and siRNAs was detected by CCK-8 assay. (C, D) An EdU assay was executed to detect the impacts of circACTN4 overexpression and knockdown on the proliferative capacity of BC cells (magnification, ×100; scale bar, 200 µm). (E) Representative xenograft tumor images of each group are shown. (F) Growth curves were plotted by measuring the tumor volume once a week. (G, H) Tumor volume and weight analyses are indicated. The data are presented as the mean ± SD, *p < 0.05, **p < 0.01.

To further assess the role of circACTN4 in BC cells, plasmid for circACTN4 over-expression and small interfering RNA against circACTN4 were constructed, and the efficiency of transfection was assessed with qRT‒PCR. The results revealed that circACTN4 was significantly knocked down or overexpressed in MDA-MB-231 and MDA-MB-453 cells (Fig. 2A). Subsequently, cell propagation and growth were investigated by knocking down or overexpressing circACTN4. EdU and CCK-8 assays revealed that elevated expression of circACTN4 conspicuously increased cell viability, whereas depletion of circACTN4 significantly suppressed the proliferative potential for BC cells (Fig. 2B–D). 1 × 10^7^ MDA-MB-231 cells transduced by circACTN4 knockdown lentiviral vectors or overexpression lentiviral vectors, and their matched control groups were subcutaneously inoculated into 4-week-old BALB/c female nude mice. The results revealed that, compared with the control, circACTN4 overexpression markedly increased the volume and weight of xenograft tumors, whereas circACTN4 knockdown obviously inhibited tumor growth (Fig. 2E–H). Collectively, these data suggest that circACTN4 could exert an oncogenic influence in the evolution of breast cancer.

### circACTN4 enhances the migration, invasion, and cell cycle progression of BC cells

**Figure 3:**
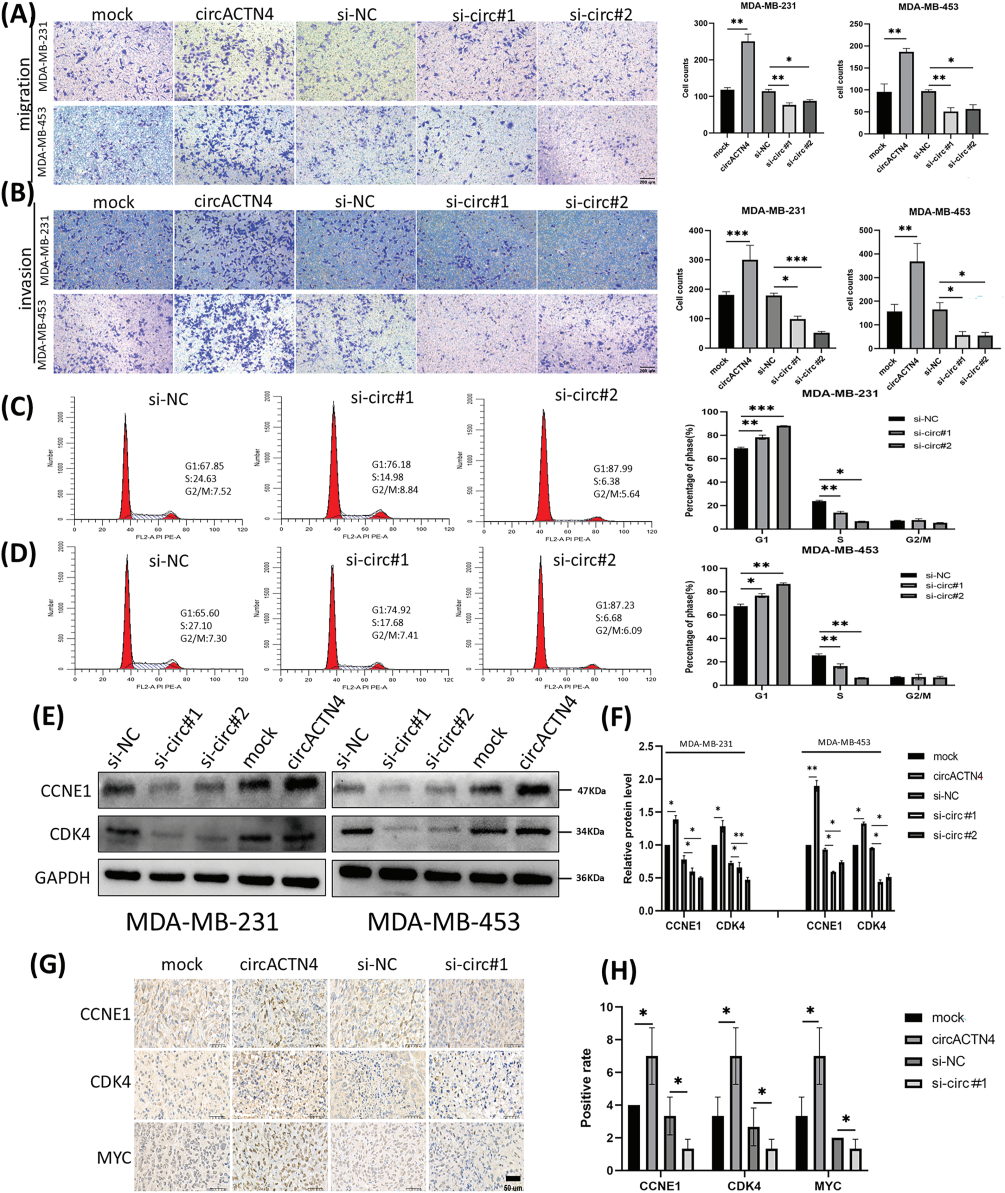
circactn4 facilitates the motility of BC cells and cell cycle progression. (A, B) The invasive and migratory potentials of BC cells were determined by transwell assay without and with Matrigel by upregulating and downregulating circACTN4 (magnification, ×100, scale bar, 200 µm). (C, D) Cell cycle analysis was performed via flow cytometry following circACTN4 depletion. (E, F) The expression of cell cycle-related proteins was examined in BC cells transfected with the indicated si-circACTN4 and circACTN4 overexpression plasmids by western blotting. (G, H) The expression levels of CCNE1 and CDK4 in tumor tissues of nude mice were assessed via immunohistochemistry, and a modified scoring method was used for the quantitative analysis of the protein expression. (magnification, ×200, scale bar, 200 μm). The data are presented as the mean ± SD, *p < 0.05, **p < 0.01, and ***p < 0.001.

To further elucidate the signaling function of circACTN4 in BC, BC cells were transfected with a plasmid engineered for the over-expression of circACTN4 and siRNAs specifically designed to target circACTN4. The invasive and migratory potentials of BC cells were notably augmented by the upregulation of circACTN4 but notably repressed by the downregulation of circACTN4, as shown by transwell assays with or without Matrigel (Fig. 3A and B). Moreover, flow cytometry was used to detect the cell cycle distribution, and knockdown of circACTN4 led to S-phase cell reduction and G1-phase cell elevation, suggesting that BC cells were arrested at G1 (Fig. 3C and D). Additionally, the levels of the cell cycle-related proteins CCNE1 and CDK4 were markedly reduced in si circACTN4 BC cells, as shown by western blotting (Fig. 3E and F). Furthermore, the impacts of circACTN4 on the MYC expression, CCNE1, and CDK4 were detected via immunohistochemistry (IHC) in transplanted tumor tissues. We found that the upregulating circACTN4 could enhance the expression level of MYC, CCNE1, and CDK4, whereas silencing circACTN4 decreased the expression of these proteins (Fig. 3G and H). The data further displayed that circACTN4 exerts an oncogenic effect on the progression of breast cancer.

### circACTN4 promotes MYC expression and AcH4 of MYC target genes

**Figure 4:**
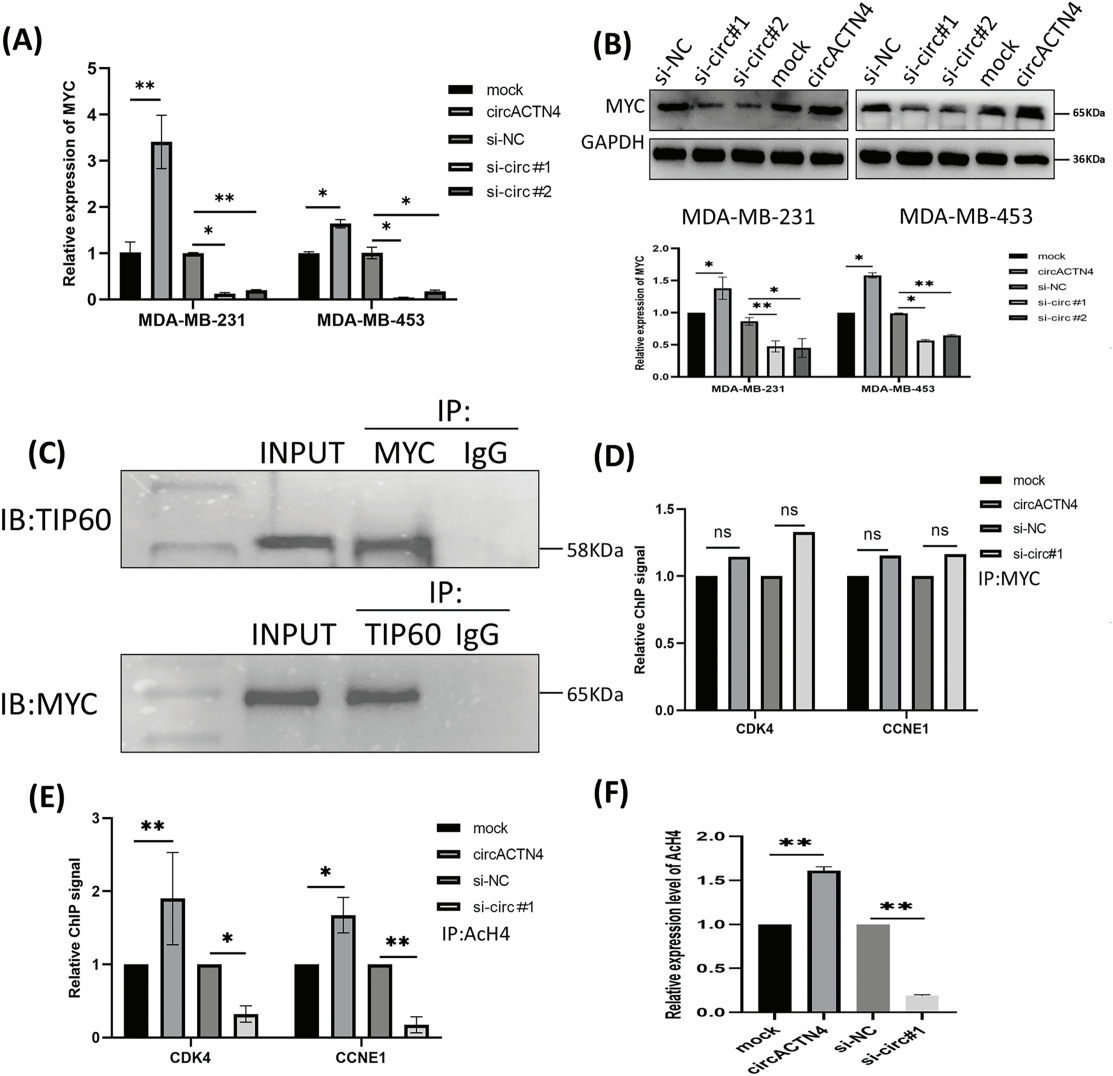
circACTN4 increases MYC expression and AcH4 of MYC target genes. (A, B) MCY expression was determined with qRT-PCR and western blotting in BC cells transfected with the indicated si-circACTN4 and circACTN4 overexpression plasmids. (C) Co-IP assays were used to detect the binding of MYC and TIP60 with anti-MYC and anti-TIP60 antibodies, respectively. (D) The ChIP-qPCR assay using anti-MYC antibodies was performed to assess the impact of circACTN4 expression on the binding of MYC to the CDK4 and CCNE1 gene promoters in MDA-MB-231 cells. (E) ChIP‒qPCR was performed with anti-AcH4 antibodies to examine the AcH4 binding signals in the promoter regions of CDK4 and CCNE1 after circACTN4 was knocked down or overexpressed in MDA-MB-231 cells. (F) Histone H4 acetylation was detected via fluorometric methods in MDA-MB-231 cells transfected with the circACTN4 overexpression construct and si-circACTN4. The statistics are displayed as the mean ± SD, ns, no significance, *p < 0.05, **p < 0.01.

To elucidate the underlying mechanism of circACTN4 in BC, MCY expression was determined by qRT‒PCR and western blotting after circACTN4 knockdown or overexpression. The results clearly demonstrated that forced expression of circACTN4 markedly upregulated MCY expression, whereas depletion of circACTN4 had the opposite effect (Fig. 4A and B). Moreover, co-IP assays were carried out with anti-MYC or anti-TIP60 antibodies, and the data indicateded that MYC could bind to TIP60 in MDA-MB-231 cells (Fig. 4C). Furthermore, a ChIP‒qPCR assay using anti-MYC antibodies revealed that the expression of circACTN4 had no effect on the binding of MYC to the CDK4 and CCNE1 gene promoters in MDA-MB-231 cells transfected with the plasmid designed for circACTN4 overexpression and siRNAs engineered to target circACTN (Fig. 4D). Next, a ChIP‒qPCR assay using anti-AcH4 antibodies demonstrated that the AcH4 binding signals in the promoter regions of the cell cycle-associated genes CDK4 and CCNE1 were significantly enhanced by the upregulation of circACTN4, whereas AcH4 enrichment in the promoter regions of CDK4 and CCNE1 was obviously reduced by the downregulation of circACTN4 (Fig. 4E). Subsequently, histone H4 acetylation was detected via a fluorometric method with an anti-AcH4 antibody. The fluorescence was measured with a fluorescence microplate reader at 530EX/590EM nm. The results demonstrated that a significant upregulation of circACTN4 increased the relative level of AcH4, while circACTN4 knockdown reduced the expression of AcH4 (Fig. 4F). The findings suggest that circACTN4 promotes the recruitment of TIP60 to MYC target genes and AcH4 through the upregulation of MCY expression. MYC might regulate the transcription of the cell cycle-related genes CDK4 and CCNE1 via indirect TIP60-mediated acetylation of histone H4 in target chromatin.

### Upregulation or depletion of MYC reverses circACTN4 knockdown- or overexpression-induced biological functions

**Figure 5:**
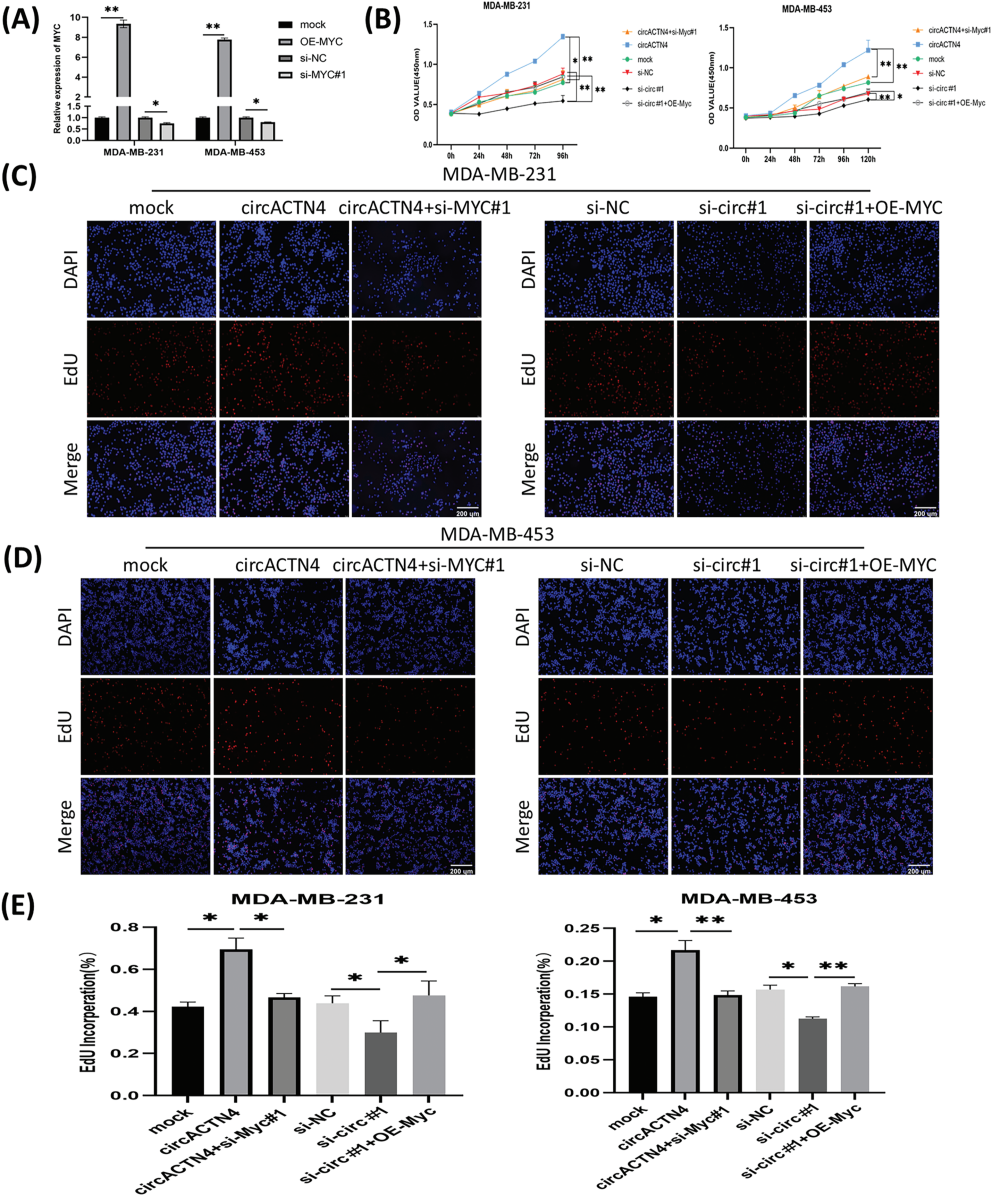
MYC overexpression or knockdown reverses the inhibitory or promotional effects on cell proliferation by downregulating or upregulating circACTN4. (A) The efficiency of the MYC overexpression vector or siRNAs in BC cells was assayed via qRT‒PCR. (B) CCK8 assays were conducted to evaluate the proliferation of BC cells transfected by the circACTN4 overexpression plasmid or si-circACTN4 and co-transfection of the circACTN4 overexpression plasmid and the si-MYC or si-circACTN4 and MYC overexpression plasmids. (C–E) EdU assays were used to determine the viability of BC cells transfected with the indicated overexpression vectors or siRNAs (magnification, ×200, scale bar, 200 μm). Data are shown as mean ± SD, *p < 0.05, **p < 0.01.

**Figure 6:**
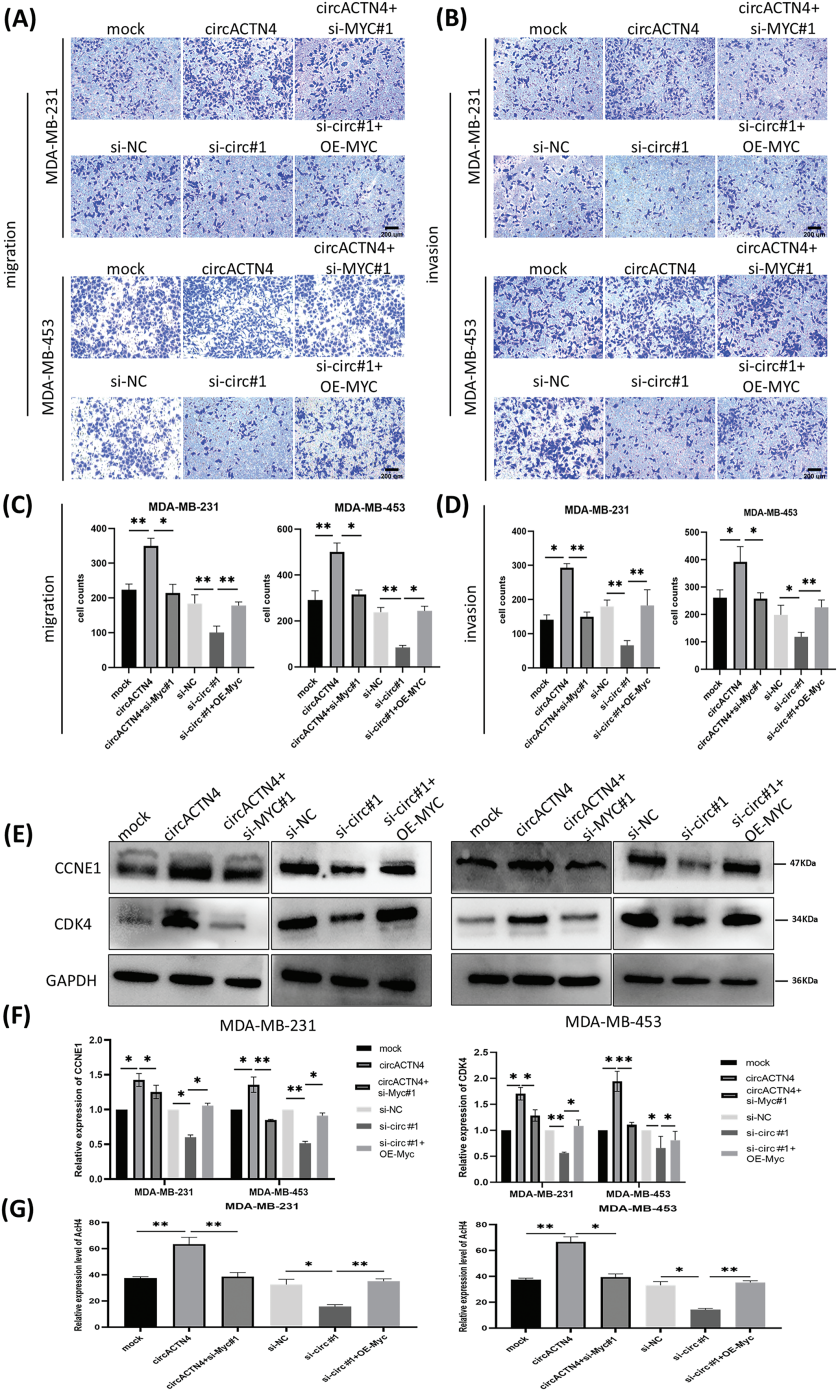
circACTN4 plays an oncogenic role via the circACTN4/MYC/AcH4 axis. (A, C) Transwell assays without Matrigel were performed to evaluate the migration ability of BC cells transfected with the circACTN4 overexpression plasmid or si-circACTN4 and co-transfection of circACTN4 overexpression plasmid and the si-MYC or si-circACTN4 and MYC overexpression plasmids. (B, D) Transwell assays with Matrigel were performed to detect the invasion ability of BC cells transfected and co-transfected with indicated overexpression vectors or siRNAs (magnification, ×200, scale bar, 200 μm). (E, F) Western blot analysis was carried out to determine the expressions of CCNE1 and CDK4 in BC cells transfected with and co-transfected with the indicated overexpression vectors or siRNAs. (G) The level of histone H4 acetylation was detected by fluorometric analysis in BC cells transfected and co-transfected with indicated overexpression vectors or siRNAs. Data are shown as mean ± SD, *p < 0.05, **p < 0.01.

To further delve into the prospective role of circACTN4 through the circACTN4/MYC/AcH4 axis, several assays were conducted in BC cells transfected with circACTN4 overexpressing plasmid or si-circACTN4 and cotransfection of circACTN4 and si-MYC or si-circACTN4 and OE-MYC. MYC overexpressing plasmid and siRNA targeting MYC were constructed, and the transfection efficiency was evaluated via qRT‒PCR. The results revealed that MYC was significantly knocked down or overexpressed in MDA-MB-231 and MDA-MB-453 cells (Fig. 5A). The data objectively indicated that MYC knockdown obviously reversed the promoting effects on the proliferation, migration, and invasion of BC cells by upregulating circACTN4, whereas the inhibitory effects of circACTN4 silencing were rescued by MYC overexpression in BC cells, as determined via CCK8, EdU and Transwell assays (Fig. 5B–E and Fig. 6A–D). Moreover, western blot analysis revealed that upregulating or downregulating MYC could antagonize the decrease or increase in CCNE1 and CDK4 expression caused by circACTN4 knockdown or overexpression (Fig. 6E and F). Histone H4 acetylation detection via fluorometric analysis revealed that MYC overexpression or knockdown reversed the decrease or increase in the relative level of AcH4, respectively, mediated by depletion or ectopic expression of circACTN4 (Fig. 6G). Together, these findings further suggest that circACTN4 promotes breast cancer cell cycle progression and oncogenesis via MYC-induced histone H4 acetylation.

## Discussion

In recent years, breast cancer has become a significant and pervasive jeopardy to the health of women across the globe. Although advances have been made in endocrine therapy, chemotherapy, and HER2-targeted therapy in recent decades, cancer recurrence and metastasis remain major challenges that urgently require innovative treatment strategies [17]. A burgeoning *corpus* of empirical evidence has corroborated that circRNAs exert essential functions in the course of tumor development and the advancement of various tumors. CircRNAs typically exhibit tissue-specific and disease-specific expression patterns, and results from rigorous previous investigations have confirmed that circRNAs hold latent clinical utility as diagnostic, prognostic, and predictive biomarkers [18].

In the present study, circACTN4 was validated to be upregulated in breast cancer, and the expression of circACTN4 was directly proportional to T stage and TNM stage and unfavorable clinical prognosis among patients with BC. Function assays revealed that circACTN4 overexpression promoted the proliferation, migration, and invasion of breast cancer cells *in vivo* and *in vitro*, whereas circACTN4 down-regulation had the reverse effects. From a mechanistic perspective, we further showed that forced expression of circACTN4 increased the expression level of MYC, whereas depletion of circACTN4 downregulated the expression level of MYC. Moreover, we demonstrated that MYC could bind to TIP60. Furthermore, circACTN4 overexpression might upregulate the expression of CCNE1 and CDK4 through increasing AcH4 levels of MYC target genes, thereby promoting cell cycle progression and tumorigenesis in breast cancer.

A substantial *corpus* of studies has corroborated that circRNAs can exert regulatory functions in sponging miRNAs, splicing genes, translating proteins, and interacting with RNA-binding proteins (RBPs) [19]. Previously, most research focused on miRNA sponges. Additionally, the interaction of circRNAs with RNA-binding proteins is also a significant function. Recent studies have shown that circRNAs could bind with RBPs and modulate the interaction between proteins and downstream target mRNAs or proteins [20]. For example, circEZH2/IGF2BP2 enhances the stability of CREB1 mRNA to promote the progression of colorectal cancer [21]. Additionally, circPTK2 can bind to PABPC1 and stabilize SETDB1 mRNA, thereby promoting SETDB1 expression, EMT, and metastasis in bladder cancer [22]. Moreover, circVPS13C interacts with RRBP1 to increase pituitary adenoma growth by reducing the stability of IFITM1 mRNA [23]. Furthermore, circDCUN1D4 interacts with HuR and inhibits glycolysis and metastasis in lung adenocarcinoma via stabilizing TXNIP mRNA [24]. Recently, circBRD7 was shown to promote the transcriptional activation and expression of its host gene BRD7 by increasing the acetylation of histone 3, thus suppressing the growth and metastasis of nasopharyngeal carcinoma [25]. In a previous study, we reported that circACTN4 might bind FUBP1 to increase MYC expression. However, further study is needed to explore the mechanism by which MYC upregulation mediated by circACTN4 regulates downstream target molecules in BC.

MYC is an important oncogene that is often overexpressed or amplified in a wide spectrum of cancers, including breast cancer. MYC regulates comprehensive gene expression and participates in cell proliferation, the cell cycle, cell differentiation, and metabolic reprogramming. MYC dysregulation is estimated to exist in approximately 70% of human cancers. MYC is adjusted at multiple levels and is a downstream effector of several signaling pathways [26,27]. Research has shown that the overexpression of MYC is associated with poorer prognosis and a greater risk of breast cancer [28]. Given the common dysregulation of the MYC oncogene in human cancer, probing the mechanism of the role of MYC in cell cycle control is important. Our previous study revealed that circACTN4 is overexpressed in breast cancer tissue and is positively correlated with the expression level of MYC. Driving cell cycle progression is the main oncogenic mechanism of the oncogene MYC. MYC can promote the cell cycle by activating cyclins and CDKs. Among the MYC targets, cyclin E/CDK2 complexes and cyclin D/CDK4 or CDK6 complexes can be activated by MYC, which is necessary for the G1/S transition of the cell cycle [29]. Consistent with previous findings, our findings indicated that circACTN4 potentially upregulates the expression level of MYC as well as its downstream effectors CDK4 and CCNE1, while circACTN4 knockdown led to G1/S arrest in BC cells.

Epigenetic dysregulation exerts a significant role in oncogenesis and development. Histone acetylation, including histone H4, is implicated in regulating chromatin structure and recruiting transcription factors to promoters of genes. Histone acetylation reduces the positive charge and affinity between histones and DNA, which results in access to RNA polymerase and transcription factors [30]. HATs and HDACs play vital roles in adjusting histone acetylation. Histone acetylation is closely related to cell cycle, proliferation, and apoptosis. Reversible H4 acetylation is commonly recognized to be associated with the potential chromatin transcription activity [31,32]. MYC acts as a transcription factor that binds to multiple promoters, regulating up to 15% of human genes. MYC recruits histone acetyltransferases (TIP60) to target chromatin and locally promotes the acetylation of histones H3 and H4 at multiple lysines [33,34]. Research has revealed that there is a positive feedback circuit between the upregulation of MYC expression, an alteration in glycolysis, and enhanced histone acetylation in Cr(VI)-transformed cells, which promotes Cr(VI)-induced cancer stem cell-like characteristics and carcinogenesis [35]. Our experimental results support these findings, and we demonstrated that circACTN4 could increase MYC expression and AcH4 levels in the target genes CCNE1 and CDK4 in BC.

TIP60 (also known as acetyltransferase 5 (KAT5)) is involved in chromatin remodeling, gene regulation, DNA repair, cancer development, and tumorigenesis by acetylating histone or nonhistone proteins. TIP60 is recruited to target promoters by various transcription factors, acetylates nucleosomal histones, and acts as a coactivator of transcription factors [36]. The function of TIP60 in cancer development is contradictory. TIP60 can activate p53 to induce apoptosis in hepatocellular cancer cells [37]. Conversely, TIP60 promoted histone H4 acetylation and cell cycle progression via MYC in B-lineage acute lymphoblastic leukemia cells [16]. Therefore, the function of TIP60 seems to be context-dependent, and its acetyltransferase activity could either promote or suppress tumorigenesis in various cancers. In the present study, we demonstrated that MYC could be associated with TIP60, suggesting that the upregulation of MYC might increase the recruitment of TIP60 to MYC target genes. Furthermore, upregulated circACTN4 increased MYC expression and AcH4 levels of the target genes CCNE1 and CDK4, thereby promoting the expression of CCNE1 and CDK4, the progression of the cell cycle, and tumorigenesis in BC. However, more detailed mechanisms involved in the regulatory process need further clarification.

## Conclusions

In conclusion, we demonstrated that circACTN4 was upregulated in BC and that a statistically robust positive association was detected between the expression level of circACTN4 and both the T stage and TNM stage a suboptimal long-term prognosis in the case of patients with BC. Moreover, upregulated circACTN4 could increase MYC expression. Additionally, we also revealed that the upregulation of circACTN4 obviously increased the proliferation, migration, and invasion of BC cells. More importantly, we found for the first time that circACTN4 is capable of augmenting the expression levels of CCNE1 and CDK4 by enhancing the AcH4 level of these genes induced by MYC, thus promoting the progression of the cell cycle and the development of BC. Our data might provide a new therapeutic target for BC and insight into the molecular mechanism of circACTN4 in the tumorigenic process.

## Data Availability

The analyzed datasets generated during the study are available from the corresponding author upon reasonable request.
